# Neuroprotective Effects of Combined Treatment with Minocycline and Olfactory Ensheathing Cells Transplantation against Inflammation and Oxidative Stress after Spinal Cord Injury 

**DOI:** 10.22074/cellj.2019.6126

**Published:** 2019-02-25

**Authors:** Soheila Pourkhodadad, Shahrbanoo Oryan, Gholamreza Kaka, Seyed Homayoon Sadraie

**Affiliations:** 1 Department of Animal Physiology, Faculty of Biology, Kharazmi University, Tehran, Iran; 2 Neuroscience Research Center, Baqiyatallah University of Medical Sciences, Tehran, Iran; 3 Department of Anatomy, School of Medicine, Baqiyatallah University of Medical Sciences, Tehran, Iran

**Keywords:** Inflammation, Minocycline, Olfactory Ensheathing Cells, Oxidative Stress, Spinal Cord Injury

## Abstract

**Objective:**

Traumatic spinal cord injury (SCI) is considered one of the most devastating injuries leading to neuronal disruption. Olfactory ensheathing cells (OECs) and minocycline have been shown to promote locomotor function after spinal cord injury. In this study, we have tested the efficacy of combined treatment with minocycline and OECs after contusive spinal cord injury.

**Materials and Methods:**

In this experimental study, adult female Wistar rats were randomly divided into five groups. Rats received an intraperitoneal injection of minocycline immediately after SCI, and then 24 hours after the injury. Transplantations were performed 7 days after the injury. Functional recovery was evaluated using the Basso, Beattie and Bresnahan scale (BBB). After that, the animals were sacrificed, and T11 segment of the spinal cord was removed after 5 weeks, and then used for histopathological, immunohistochemical, and biochemical assessments. Western blot analysis was applied to determine the protein expression of tumor necrosis factor alpha (TNF-α), interleukin 1 beta (IL1β) and caspase3.

**Results:**

The results of this study showed that the combination of OECs graft and minocycline reduced the functional deficits and diminished cavitation and astrogliosis in spinal tissue. The analysis of protein expression by western blotting revealed that minocycline treatment along with OECs transplantation further decreased the level of IL-1β, TNF-α, caspase-3, and the oxidative stress as compared with when minocycline or OECs transplantation was used alone.

**Conclusion:**

The combinatory treatment with OECs graft and minocycline induced a more effective response to the repair of spinal cord injury, and it is considered a therapeutic potential for the treatment of SCI.

## Introduction

Spinal cord injury (SCI) is considered one of the most devastating conditions leading to neurological dysfunction and disability in young people (1). Traumatic SCI which is resulted in functional deficits causes degeneration and disruption of axonal tracks leading to secondary injury and cell death that occur hours and days after the primary trauma (2, 3). It is thought that inflammation, the oxidative stress, and apoptosis are significant factors precipitating in post-traumatic degeneration due to secondary injury in SCI. Although the molecular pathway of secondary damage is still controversial, therapeutic strategies that inhibit and delay oxidative stress and apoptosis may contribute to motor functional recovery (4, 5). 

Minocycline, a semi-synthetic second-generation tetracycline, has several mechanisms of action including anti-inflammatory (6) and anti-apoptotic effects (7). It also reduces the microglial activation made it an attractive neuroprotective agent (8). Many studies indicated that minocycline exerts neuroprotective effects in several rodent models of the central nervous system disorders including ischemia, Huntington’s disease, amyotrophic lateral sclerosis, and spinal cord injury (6, 9, 10). In another experiment, it has been revealed that minocycline provides neuroprotection against 6-hydroxydopamine or glutamate-induced toxicity by inhibiting microglial activation (11, 12). These experimental studies demonstrate that minocycline provides neuroprotection via an anti-inflammatory mechanism that may help the survival of transplanted cells. 

Numerous investigators sought strategies to promote axonal regeneration following SCI, and cellular transplantation has been emerged as a promising tool to achieve this goal. Among cellular manipulation strategies, olfactory ensheathing cells (OECs) have attracted much attention as potential therapeutic agents for the treatment of SCI due to their ability to secrete neurotrophic factors and remyelinate the regenerated axons (13, 14). Despite the transplantation of OECs after SCI has been successful so far, the functional recovery after the injury is achieved only to a partial degree (15). To date, the underlying mechanism of SCI is complex, and many factors are involved in the development of the disease. Although the application of OECs has opened up a new horizon for the treatment of neurodegenerative diseases, it is not useful for spinal cord repair in animal models when employed alone. Thus combined therapies are recommended to boost the efficacy of this therapeutic approach. The previous studies reported the transplantation of OECs in addition to the administration of FK506 and methylprednisolone. However, the restoration of functions was not achieved completely post- injury (16, 17). According to former studies, minocycline and OECs transplantation have been indicated to possess suitable effects on SCI. Thus, the aim of this study was to determine whether the restorative properties of OECs graft is improved when combined with minocycline administration after spinal cord contusion injury. 

## Materials and Methods

In this experimental study, adult female Wistar rats (220250 g) were used in this study. The animals were maintained on a 12 hours dark/light cycle at 20°C. Food and water were available ad libitum. All procedures that pertained to animals were approved by the animal care and ethics in Baqiyatallah University of Medical Sciences, Tehran, Iran. For inducing SCI, we used 50 rats in the following five groups (10 rats in each group): sham group in which only laminectomy was performed; control group in which the animals underwent laminectomy, SCI, and the phosphate-buffered saline (PBS) treatment (i.p) following the transplantation of Dulbecco’s Modified Eagle’s medium (DMEM) into spinal cord 7 days post-injury; OECs group in which the animals underwent laminectomy, SCI, and the PBS treatment followed by the transplantation of OECs (450000 cells/6 µl) at 7 days post- injury; minocycline group in which the animals underwent laminectomy, SCI, and the minocycline treatment (90 mg, i.p, given the first and 24 hours after SCI) followed by the transplantation of DMEM (6 µl) into the spinal cord at 7 days post-injury, and finally, OECs+minocycline group in which the animals underwent laminectomy, SCI, and minocycline treatment (90 mg/kg, i.p) followed by the transplantation of OECs (450000 cells/6 µl) at 7 days post-injury. We also used 10 rats for OECs culture. 

### Olfactory ensheathing cells culture and immunopurification 

OECs were obtained from the nerve fibers and olfactory bulbs of adult rats using Nash methods (18). Briefly, rats were anesthetized with an overdose of chloral hydrate, then, the olfactory nerve rootlets and olfactory bulbs were dissected and placed into calcium and magnesium-free Hank's balanced salt solution (HBSS, Sigma, USA). All meninges and blood vessels were divested of the tissue. The tissues were minced and incubated within a solution of 0.1 % trypsin (Gibco, USA) in DMEM/F12 (Gibco, USA) in 5% CO_2_ at 37°C for 30 minutes. Trypsinization was inactivated by the addition of fetal bovine serum (FBS, Sigma, USA). The suspension was centrifuged at 1000 rpm for 5 minutes and seeded into an uncoated cell culture flask in DMEM/F12 (Gibco, USA) supplemented with 10% fetal bovine serum, 2 Mm L-glutamine (Gibco, USA), 100 IU/ml penicillin and 100 µg/ml streptomycin (Gibco, USA), a process allowing most of the fibroblasts to attach to the plate during the first incubation period for 18 hours. The supernatant from the culture was removed and plated onto uncoated culture flasks. After 36 hours of incubation, the supernatant was seeded in flasks precoated with poly L-lysine (Sigma, USA), and the OECs attached within 48 hours. The media were changed every 2 days. After reaching confluence, OESc were identified by immunohistochemistry (IHC) staining with p75 nerve growth factor receptor (NGFRp75) antibody (1:100, Rabbit polyclonal, N3908, Sigma, USA) to determine cell purity. 

### The animal model of spinal cord injury 

Rats were anesthetized with intraperitoneal chloral hydrate (450 mg/kg). A laminectomy was done at vertebral level T11, and the spinal cord was exposed. The injury was produced by dropping a 10 g rod from a height of 25 mm onto the rat spinal cord at T11, following the procedural guidelines established by a multicenter consortium. After the injury, the muscles and skin were closed separately, and the rats were placed in a chamber overnight. Gentamicin was administered for 3 days after contusion to prevent wound and bladder infections; also, acetaminophen was added to drinking water for 7 days, and urinary bladder expression was performed twice daily until reflexive bladder emptying was achieved.

### Minocycline administration

Minocycline was dissolved in sterile PBS and administered intraperitoneally (i.p) after injury in the treatment group. Rats receiving 25 mm insult received 90 mg/kg of minocycline immediately after SCI, and then 24 hours after SCI (19). The control group received an injection of sterile PBS. For the sham groups, the animals underwent T11 laminectomy without contusion injury, received non-pharmacological treatment, and were sacrificed at the same time intervals as the treatment groups. 

### Transplantation

The transplantation was performed 7 days after the initial surgery (14). All rats were anesthetized, and the laminectomy site was re-exposed. Six microliters of cell suspension (450,000 cells/6 µl for OECs) were injected using a Hamilton syringe, which remind in place after each injection for 5 minutes. The cell suspension was injected at a depth of 0.8 mm of the lesion epicenter and 1 mm rostral and caudal to the epicenter (2 µl per injection). Control animals were injected with an equal volume of DMEM at the same sites. After injection, the muscle and skin were sutured. 

### Behavioral assessment

Behavioral tests were performed according to theBasso, Beattie and Bresnahan scale (BBB scale) to evaluate the functional recovery (20). The scaleused for measuring hind limb function rangedfrom 0 (paralysis) to 21 (normal score), with an increasing score indicating the use of individual joints, coordinated joint movement, coordinated limbmovement, weight-bearing, and the other functions. All scores were obtained on days 1, 7, 14, 21, 28, and35 by two examiners who were blinded by treatment. The average scores were calculated according to the progression of locomotion recovery after SCI.

### Histological and immunohistochemical analyses 

The spinal cord segment at the level of T11 was dissected(1 cm on each side of the lesion) 35 days after SCI, andthen, were paraffin embedded and cut into 5 µm-thick transverse sections by a microtome. Sections were then deparaffinized with xylene, rehydrated with decreasing alcohol concentrations, then stained with hematoxylin and eosin (H&E). Cavity volume in all sections was studied using an image analyzing software (Motic 2.1, Italy, Cagli). The transverse sections were stained with a primary antibody against the glial fibrillary acidic protein (Rabbit anti-GFAP, 1:100; PAB12325; Abnova, Taiwan) to visualize the astroglial reactivity and the formation of glial scar around the lesion. Segments of the spinal cord centered on the impact site were cut into serial 5-µm-thick sagittal sections for histopathology, (n=3 rats/group). The sections were permeabilized and blocked with 0.3 % Triton X-100 and 10% normal goat serum in 0.01 M PBS for 2 hours. Then sections were incubated at 4°C with polyclonal rabbit anti-glial fibrillary acidic protein (GFAP, 1:100) for astrocytes in a wet chamber overnight. After washing with PBS 4 times, the sections were incubated with HRP-conjugated secondary antibodies (1:200; Abnova, Taiwan) for 2 hours at room temperature. After incubationwith 0.02% 3,3’-Diaminobenzidine (DAB) for 5 minutes, thesections were counterstained with hematoxylin. The positivearea counting was performed in a defined square perimeterof 1,000 µm^2^ in three different segments of the ventral horn.

### Western blot assay 

Western blot was used to detect the protein expression of tumor necrosis factor alpha (TNF-α), interleukin 1 beta (IL1ß), and caspase-3. After being treated with the transplantation of OECs and minocycline for 35 days, 5 mm lengths of the spinal cord centered on T11were rapidly removed, weighted, and the tissues were homogenized in 0.2 mL of homogenization buffer; then, centrifuged for 10 minutes (12,000 rpm/minutes, at 4°C). The supernatants were applied for protein determination. 20 µg protein samples were separated by 10% sodium dodecyl sulfate-polyacrylamide gel electrophoresis (SDS-PAGE) and transferred from the gel onto polyvinylidene fluoride (PVDF) membranes (150 mA, 1.5 hour) (Millipore Corporation, USA). After blocking with 5% nonfat dry milk for 2 hours, the membranes were incubated overnight at 4°C with different primary antibodies including anti-TNFa (Abcam, Cambridge, UK), anti-IL1ß (Abcam, Cambridge, UK), anti-caspas3 (Abcam, Cambridge, UK), and anti-GAPDH (Abcam, UK). After washing membranes with TBST, the membranes were incubated with goat anti-rabbit IgG- HRP conjugated secondary antibody (Sigma, USA) at a 1:1000 dilution for 2 hours at room temperature. Then, the membranes were rinsed three times for 10 minutes and incubated with enhanced chemiluminescence (ECL) kit. GAPDH served as the internal control, and the analysis of the images was performed using the ImageJ software.

### Tissue preparation and protein quantification

At 35 days after SCI, the spinal cord tissues were removed and homogenized in cooled radioimmunoprecipitation assay (RIPA) buffer supplemented with phenyl methanesulfonyl fluoride, then centrifuged at 15,000 × g for 15 minutes at 4°C. Next, the supernatant was aliquoted and stored at 20°C until used for the measurement of the oxidative stress parameters analysis. The concentration of protein was measured using the Lowry method (21). 

### Measurement of tissue malondialdehyde 

The concentration of malondialdehyde (MDA) was determined based on its reaction with thiobarbituric acid (TBA) at 95°C (15). Briefly, 150 µl supernatant was mixed with 300 µl trichloroacetic acid (10%, Sigma, USA) and TBA (0.67%, Sigma, USA) and heated at 95°C for 15 minutes. After cooling at room temperature, the samples were centrifuged at 3500 ×g for 10 minutes. The absorbance of the samples was read at 532 nm. Tetramethoxypropane (Sigma, USA) was used to prepare the standard curve. The malondialdehyde (MDA) concentrations were reported as nmol/mg protein. 

### Measurement of catalase activity 

Catalase activity was calculated according to the method of Aebi (22). The reaction was started by the addition of tissue homogenate (50 µg) in 2 ml of 30 mM hydrogen peroxide (H_2_O_2_) in 50 mM phosphate buffer (pH=7.0). The activity was measured by the reduced absorbance of H_2_O_2_ at 240 nm. The results are expressed as units per mg of protein (U/mg of protein). 

### Levels of the glutathione

Glutathione (GSH) levels were determined based on the reaction between dithionitrobenzoic acid (DTNB) and the reduced GSH. The yellow mixture was measured spectrophotometrically at 412 nm. GSH content was expressed as mg GSH/g protein.

### Nitrite oxide assay (nitrite content)

To measure tissue levels of nitrite oxide (NO) in spinalcord samples, 50 µL of supernatant was mixed with an equal volume of Griess reagent (1% sulphanilamide and 0.1% N-1naphthylethylene diamine dihydrochloride in 0.5% H_3_PO_4_). After incubation for 10 minutes at room temperature, the absorbance was measured at 540 nm in a microplate reader (23). The average concentration of nitrite was calculated through a comparison with a standard calibration curve with sodium nitrite (NaNO_2_: 0-110 µmol/l).

### Statistical analysis

All data are expressed as the mean ± SEM and were analyzed using the GraphPad Prism software, version 5.0 (GraphPad Software, Inc., La Jolla, CA, USA). The statistical differences were determined using one-way analysis of variance (ANOVA) with post-hoc Bonferroni’s multiple comparison tests. Differences were considered significant if P<0.05. 

## Results

**Fig.1:**
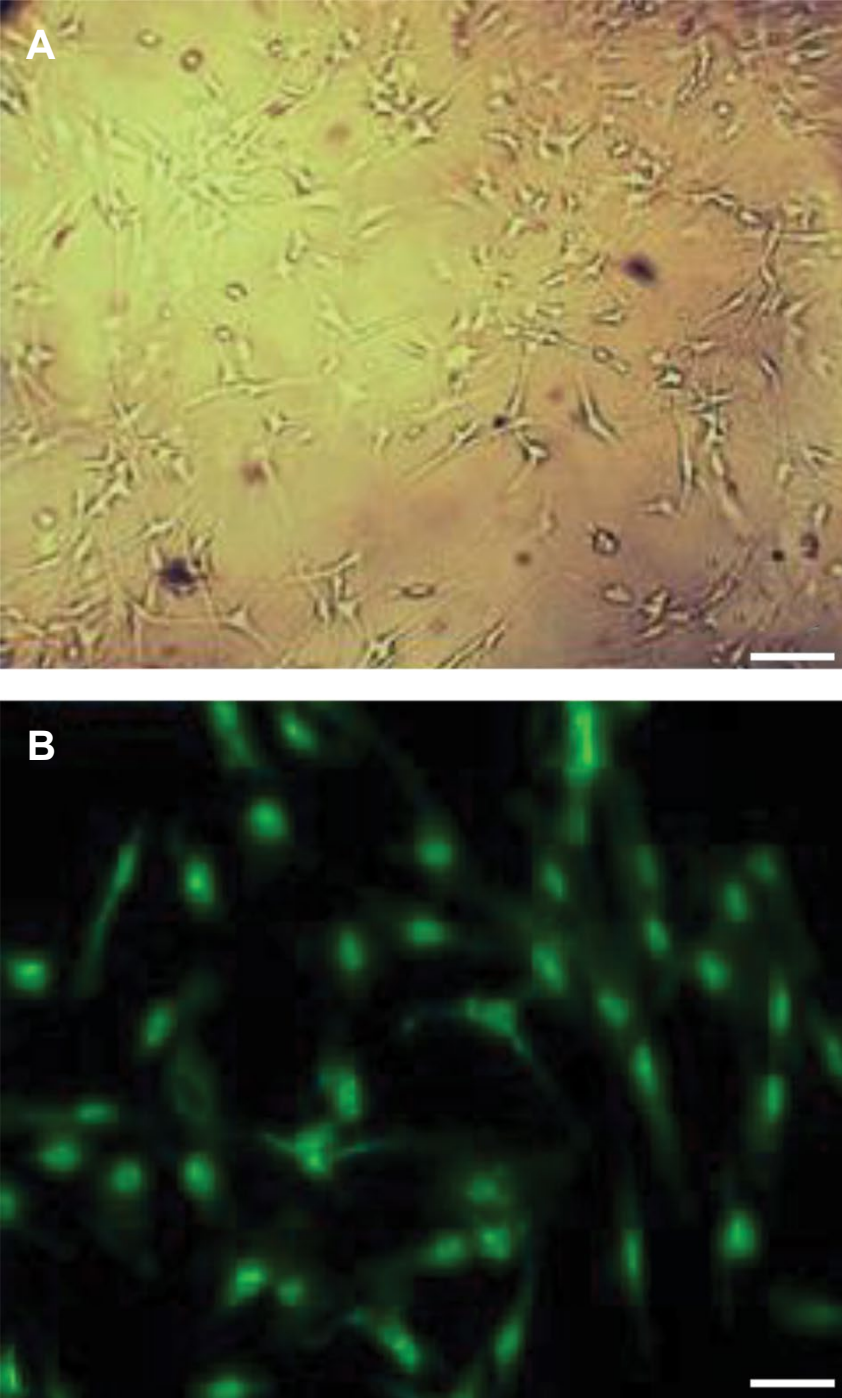
Characterization of primary cultured olfactory ensheathing cells (OECs). A. The morphology of OECs in culture and B. Immunofluorescence analysis of NGFRp75 (shown in green) in the cells. The purity of OECs is 85% (scale bar: 100 µm).

A week after the cells were plated, various forms of classic cells of OECs were observed under microscopy as bipolar and multipolar cells (Fig .1A). To identify OECs, immunocytochemical staining was utilized for the detection of NGFRp75 (Fig .1B). 

### Locomotor recovery

**Fig.2:**
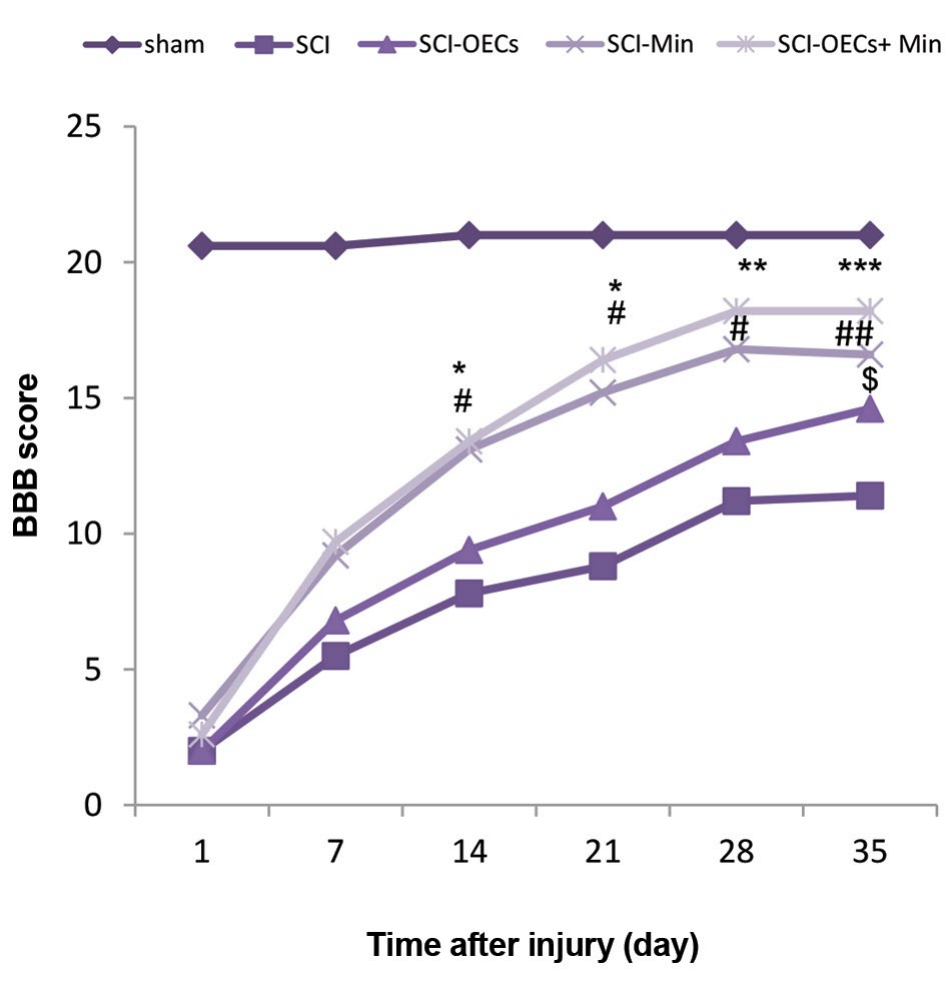
Effect of combination therapy on hind limb behavioral motor function after SCI. Data are expressed as the means ± SEM. SCI; Spinal cord injury, OECs; Olfactory ensheathing cells, BBB; Basso, Beattie and Bresnahan scale, *** ; P<0.001, **; P<0.01, *; P<0.05 in SCI-OECs-Min group versus SCI group, #; P<0.05, ##; P<0.01 in SCI-Min group vs. SCI group, and $; P<0.05 in SCI-OECs group versus SCI group.

The locomotor behavior for both hind limbs was impaired in all groups immediately after contusion injury. The motor function of the four groups exhibited gradual improvements in the hind limb during 35 days of the experiment. Although motor functions were gradually improved, the scores of motor function were significantly lower (P<0.001) than those of the sham group. Similarly, an improved motor function was also found in the minocycline treatment on day 14, 21, 28 (P<0.05), and 35 (P<0.01) and in the OECs transplantation group on day 35 (P<0.05) as compared with the SCI group. The combined treatment group showed a markedly better functional recovery, with a significantly increased BBB locomotor score on day 14, 21 (P<0.05), 28 (P<0.01), and 35 (P<0.001) compared to the SCI group (Fig .2). 

### Cavitation analysis

**Fig.3:**
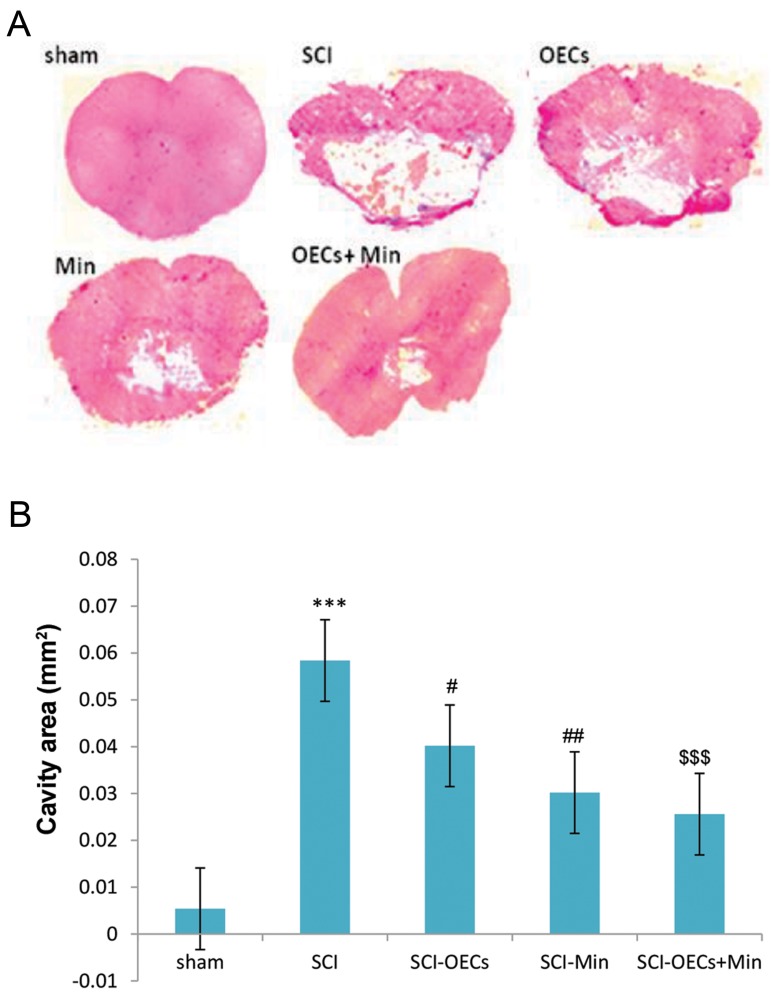
Histopathological assessment of combined treatment with OECs and minocycline on the cavity area at the epicenter of injured spinal cord. A. The H#E stained paraffin sections of cavity area (×10) and B. Percentage of the cavity area at the epicenter of injury between injury groups at 35 days after SCI. Data are presented as mean ± SEM. SCI; Spinal cord injury, OECs; Olfactory ensheathing cells, *** ; P<0.001 versus sham group, #; P<0.05, ##; P<0.01, and $$$; P<0.001 versus SCI group.

The mean cavity size was calculated after H&E staining. At 35 days after injury, the SCI control group showed a maximum injury and minimum recovery from SCI, and severe tissue damage was observed in the gray and white matter. In the sham group, the white and gray matter of the spinal cord segments were intact (Fig .3A). 

The results indicated that the mean cavity size was significantly lower in the minocycline- and OECstreated groups in comparison with the SCI group (P<0.01, P<0.05). Although the percentage of the cavitation in the OECs transplantation group showed a slight decrease compared to the minocycline group, the difference was not statistically significant (P>0.05). Moreover, the mean cavity area in the minocycline+OECs group was significantly reduced in comparison with the SCI (P<0.001, Fig .3B). 

### Effects of combined treatment with minocycline and olfactory ensheathing cells transplantation on GFAP after spinal cord injury

**Fig.4:**
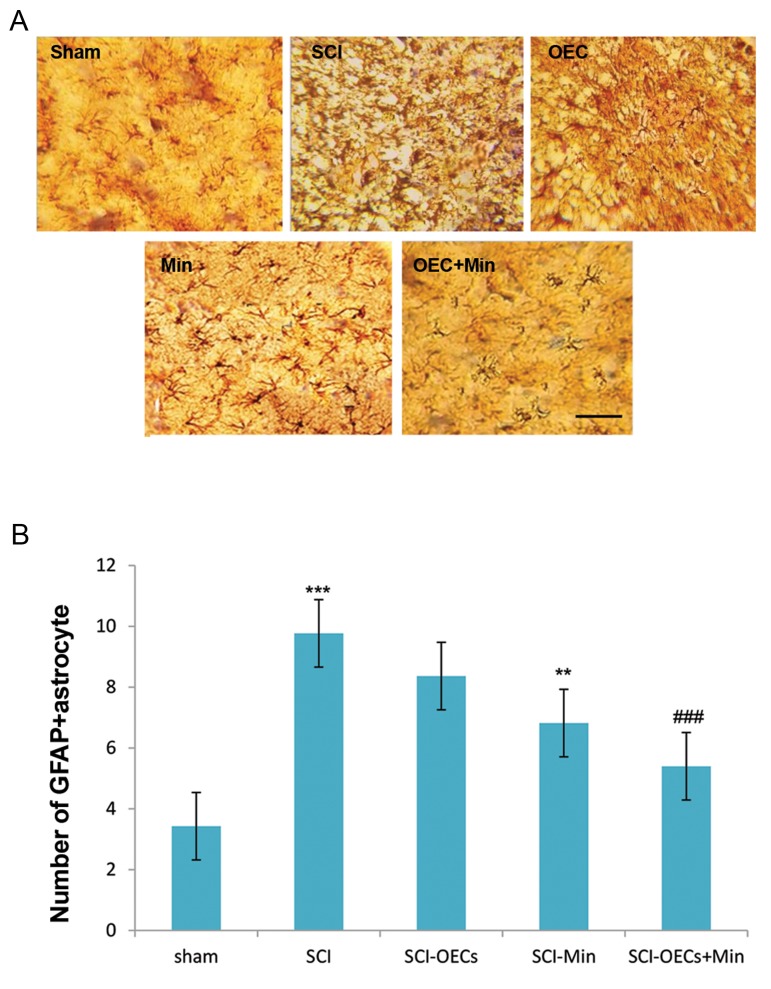
Immunohistochemistry assessment of combined treatment on the GFAP in the ventral horn of spinal cord at 35 days after SCI. A. The immunohistochemistry staining (×40) (scale bars: 50 µm) and B. Number of the GFAP-positive glial. Data are presented as the mean ± SEM. GFAP; glial fibrillary acidic protein, SCI; Spinal cord injury, OECs; Olfactory ensheathing cells, *** ; P<0.001 vs. sham group, **; P<0.01, and ###; P<0.001 vs. SCI group.

To identify whether the different treatment groups inhibited posttraumatic astrogliosis, the GFAP expression was compared between experimental groups. There was strong, robust immunoreactivity in the grey matter throughout all sections of the SCI group. The statistical analysis revealed that the number of GFAP+ astrocytes was significantly increased in the SCI group. Nevertheless, this activation was significantly attenuated in the minocycline and minocycline+OECs groups, whereas the OECs group had intermediate values. Regarding the obtained results, the density of astrogliosis in the gray matter of the spinal cord was significantly increased in the SCI group in comparison with the sham group (P<0.001). Moreover, the statistical analysis showed that the density of gliosis was significantly reduced in the minocycline+OECs (P<0.001), and minocycline (P<0.01) groups when compared with the SCI group (Fig .4A, B). There were no significant differences between the OECs and SCI groups. 

### Effect of combined treatment with minocycline and olfactory ensheathing cells transplantation on expression levels of pro-inflammatory factors after spinal cord injury 

**Fig.5:**
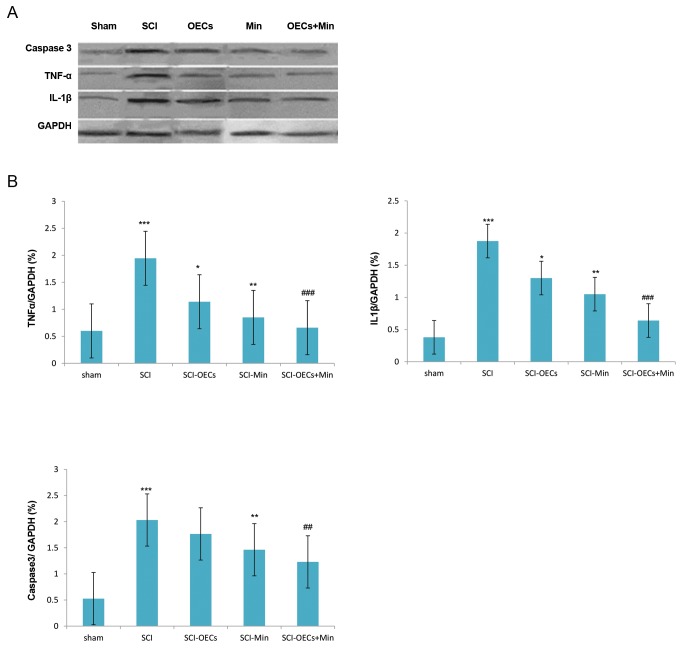
The effect of combined treatment on the levels of TNF-α, IL-1ß and caspase-3. A. Western blotting for TNF-α, IL-1ß and caspase-3 in different groups and B. The quantification of protein expression of TNF-α, IL-1ß, and caspase 3 at 35 days after SCI. Data are presented as mean ± SEM (n=4, each). TNF-α; Tumor necrosis factor alpha, IL-1ß; Interleukin 1 beta, ***; P<0.001 vs. sham group, *; P<0.05, **, ##; P<0.01, and ###; P<0.001 vs. SCI group.

The expression of proinflammatory factors was also determined to elucidate the functions and mechanisms of inflammatory cells. The analysis of protein levels by western blotting revealed that minocycline treatment and OECs transplantation significantly decreased the level of IL-1ß, TNFa, as compared with that of the SCI group (P<0.01, P<0.05, Fig .5A, B). Also, the results showed that the transplantation of OECs with minocycline reduced the levels of IL-1ß and TNF-α (P<0.001, Fig .5A, B). These results suggested that the transplantation of OECs with minocycline can reduce further the expression of pro-inflammatory factors (TNF-α and IL-1ß) in SCI.

### Effects of combined treatment with minocycline and olfactory ensheathing cells on caspase-3 activation after spinal cord injury 

Western blot analysis was used to detect the expression of caspase-3 in the spinal cord tissue at 35 days after SCI. In comparison to the sham group, the expression level of caspase-3 was significantly elevated after SCI (P<0.001). Nevertheless, minocycline and combined treatment with minocycline and OECs significantly decreased SCI-induced increase in caspase-3 activity (P<0.01). However, the transplantation of OECs had no significant effect on the expression of caspase-3 (Fig .5). 

### Biochemical findings

**Fig.6:**
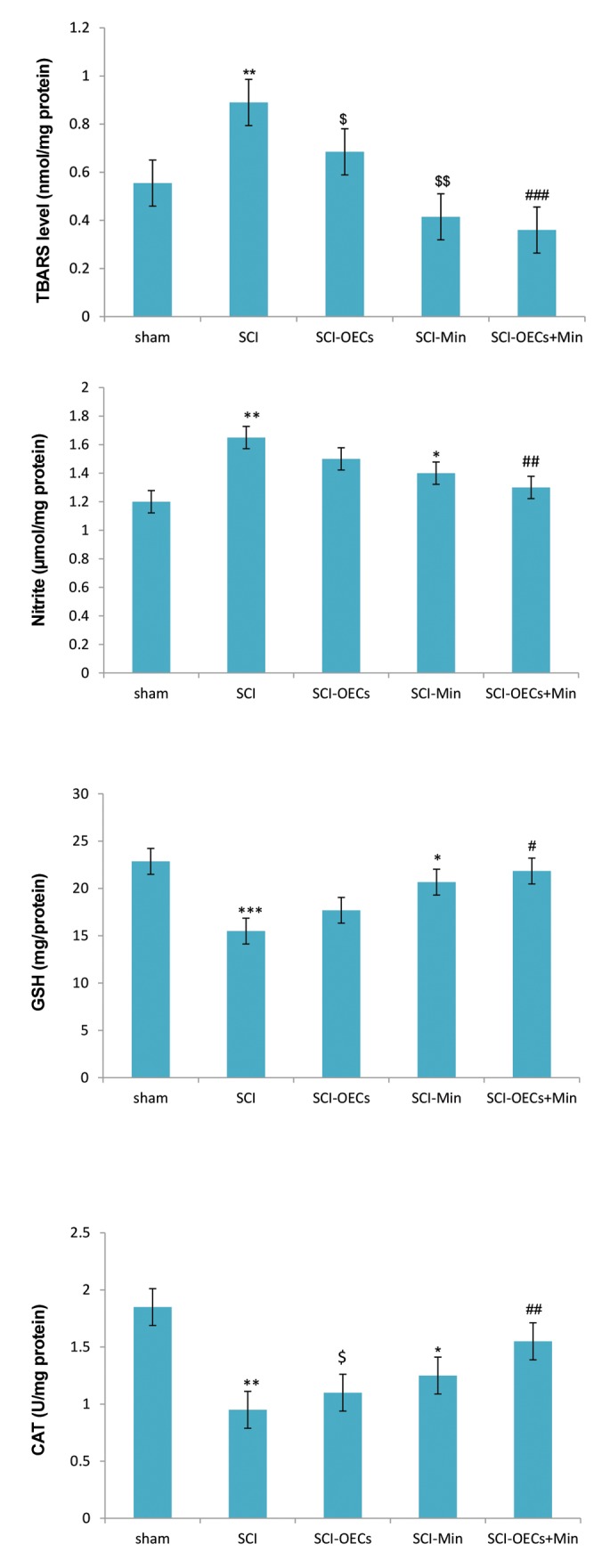
The effect of combined treatment on the levels of MDA, NO, CAT, and GSH at 35 days after SCI. The error bars indicate mean ± SEM. MDA; Malondialdehyde, NO; Nitric oxide, CAT; Catalas, GSH; Glutathione, SCI; Spinal cord injury, OECs; Olfactory ensheathing cell, ** ; P<0.01, ***; P<0.001 vs. sham, $$; P<0.01, *; P<0.05, $; P<0.05, ###; P<0.001, ##; P<0.01, #; P<0.05 vs. SCI group (n=6/group).

The levels of GSH and CAT were significantly lowered in the SCI control animals compared to the sham group (P<0.001, P<0.01). The OECs transplantation had no significant effects on GSH activity when compared to the SCI group, but it increased the levels of CAT (P<0.05). However, SCI animals treated with minocycline and combined treatment with the minocycline+OECs exhibited a significant ameliorating effect on the level of GSH compared to the SCI group (P<0.05, Fig .6). Both treatment with minocycline and minocycline+OECs significantly increased the tissue CAT activity compared to the SCI group (P<0.05, P<0.01, Fig .6). 

The results of TBARS indicated that SCI significantly stimulated the level of TBARS activity compared to the sham group (P<0.01). However, SCI animals treated with either OECs or minocycline alone, or in combination with each other were significantly mitigated compared to the SCI group (P<0.05, P<0.01, P<0.001). Tissue NO levels were found to be significantly increased in the SCI group when compared with the sham group (P<0.01). In the minocycline and combined treatment groups, tissue NO levels were significantly decreased compared to the SCI group (P<0.05, P<0.01). In the OECs but didn’t show significant difference in the NO levels compared to the SCI group (Fig .6). 

## Discussion

The secondary injury after SCI leads to significant loss of neurons and the formation of an inhibitory glial scar. A variety of single therapies have targeted single obstacles that limit the recovery of post-injury, which provide small improvements in functional recovery (24). Earlier studies have indicated that axonal regeneration in SCI is possible if the inhibitory milieu or glial scar is prevented at a low level to allow CNS axons to grow (25, 26). Herein, we combined promising therapies namely, transplantation of OECs and minocycline to overcome the multitude of obstacles limiting the recovery with the aim of enhancing recovery over single therapies. Also, in this study, for the first time, we investigated the effect of OECs alone and in combination with minocycline on the oxidative stress in contusive SCI model. The results of this study indicated that the effect of combined treatment with OECs and minocycline on biochemical factors and apoptosis is more effective than single treatment with OECs or minocycline. 

The results showed that the combination of minocycline with OECs grafting results in a significant improvement in BBB score than the SCI group, also an increase in tissue sparing observed in the combination of minocycline and OECs transplantation compared to minocycline and OECs transplantation alone. OECs transplantation after moderate contusive thoracic SCI of adult rats promoted the partial recovery of motor function that is in agreement with the study of Plant et al. (14). The most recovery rate was apparent in the minocycline and minocycline+OECs groups, which exhibited the improvement in the functional recovery with an increased rate of recovery between 2-5 weeks after SCI. This may be explained by this fact that the injection of minocycline prior to OECs transplantation provides a favorable environment for grafted cells by reducing proinflammatory molecules and glial scar formation. On the other hand, GFAP expression is increased during the first week of spinal cord injury; therefore, OECs grafting, one week after injury, may be too delayed to prevent the formation of the glial scar and secretion of inhibitory molecules. In one study performed by López-Vales et al. (15) showed that the delayed OECs transplants had intermediate effects on the GFAP expression after SCI. Therefore, the protective effects after contusion SCI and the enhanced locomotor function were observed when the combination of minocycline and OECs transplantation was applied that may mediate the inhibition of the posttraumatic astrogliosis. These findings are in agreement with the results of similar studies. Festtof et al. in 2006 reported that modulating apoptosis, caspases, and microglia by minocycline provide promising therapeutic targets for limiting the degree of functional loss after CNS trauma (9). Besides, neuroprotection effect of minocycline has been also reported to promote axonal regeneration through the suppression of RGMa in rat MCAO/reperfusion model (27). Consistent with these studies, we indicated that minocycline enhanced the functional recovery after moderate contusive spinal cord injury. On the other hand, the results observed the restorative effects of OECs transplant after SCI that are in agreement with previous studies (28-30). 

Also, the histological results indicated that the cavitation volume in animals receiving minocycline was significantly reduced as compared with those received OECs graft. It was shown that the minocycline group had the increased volume of tissue sparing 35 days post- injury, but the combination of OECs transplantation with minocycline further reduced the cavity size compared with the single strategies. Furthermore, the combination therapy was more effective in increasing the tissue sparing than minocycline and OECs transplantation alone. These results would be expected due to the difference in the timing of the injection of minocycline immediately after the injury, during the peak of secondary injury, versus transplantation of OECs one week after injury when considerable secondary tissue loss had already occurred. Moreover, the secondary injury was increased because of the delayed treatment, and the injury cascade that stems from the neurodestructive events is likely to be more extensive. Besides, the immunomodulatory effect of minocycline was exerted through the protection of the spinal cord tissue and reduced neuronal and glial death during the acute phase of the injury, such as inhibition of caspase-3 activity (9) and the release of cytochrome c from mitochondria (10). Lee et al. (31) indicated that minocycline reduces neuronal death and the cyst cavity, and it improves the locomotor function after traumatic SCI in rats. On the other hand, tissue protection mediated by OECs is due to the ability of OECs in secretion of several factors that may promote not only axonal regeneration but also provide neurotrophic support that permit the survival of the damaged neural cells, including nerve growth factor, brain-derived neurotrophic factor, glial derived neurotrophic factor, and neurotrophin 4/5 factor, as well as the prevention of the progression of cavity (32). Because each treatment modulates some common factors involved in the pathophysiology of SCI through the different mechanisms; therefore, the combination of tissue protective agents and the later transplantation of cell may exert additive tissue sparing over the use of each treatment alone. 

It was previously reported that the reactive astrocytes secrete cytotoxic proinflammatory factors and chondroitin sulfate proteoglycans that initiate the effective cascades, which not only increase the inflammatory responses but also destroy the internal environment of the CNS resulting in cell death and inhibition of the axonal regeneration (33, 34). Thus, reducing the levels of pro-inflammatory factors can prevent the subsequent cytotoxic and apoptotic effects. Herein, in accordance with the others studies, we demonstrated that both minocycline injection and OECs transplantation reduced proinflammatory cytokines such as TNF-α and IL-1ß. However, OECs treatment did not decrease the expression of caspase-3 after SCI. Nevertheless, the combination of both treatments further reduced proinflammatory cytokines and caspase-3 in the contusion SCI model. 

In the present study, lipid peroxidation measured as thiobarbituric acid-reactive substances in tissue (MDA), NO, and ROS levels as an indicator of oxidative damage were analyzed for the mechanisms underlying the neuroprotective action of OEC grafts for the first time in SCI. The previous studies have reported that the transplanting of OECs into the sub-retinal space of rats with light-induced retinal damage reduced the oxidative stress and the loss of photoreceptors (35). Also, in another study, it was shown that OEC-conditioned medium may also promote the antioxidant defense, leading to suppression of 6OHDA-induced oxidative damage by enhancing Akt survival signaling (36). A study carried out by Liu et al. (37) indicated that OEC-conditioned medium may protect astrocytes from the oxidative damage by promoting the cell survival while reducing apoptosis of the damaged cells.

In the present study the levels of MDA, and NO were significantly increased following SCI. In addition, due to elevated levels of the oxidative stress in the spinal cord, tissue antioxidants namely GSH and CAT were decreased (38). Minocycline and OECs alone and in combination with each other significantly decreased the levels of MDA, and NO when compared with the SCI group. These results have shown that OECs transplantation one week after injury could affect the oxidative stress and proinflammatory factors. However, the underlying mechanisms of the protective effect of OECs have not been fully understood and need further studies. These results suggest noticeable protection against the oxidative stress and significant antioxidant effect of combined treatment in rats with contusive spinal cord injury. Similarly, Ahmad et al. (39) also reported that minocycline treatment decreased tissue MDA and MPO levels and prevented the inhibition of GSH and CAT in SCI tissues. Furthermore, other studies have determined that minocycline potentially targets a broad range of secondary injury mechanisms, and protect neural tissue from multiple neurotoxic insults via its anti- inflammatory, anti-oxidant, and anti-apoptotic properties as well as inhibitory impacts on lipid peroxidation and oligodendrocyte apoptosis. It was demonstrated that the treatment with minocycline improved the functional recovery after SCI (40).

## Conclusion

The results of the present study showed that minocycline and OECs grafts can modulate some common mechanisms involved in the pathophysiology of spinal cord injury, and therefore, the combination of both treatments may exert better effects. The injection of minocycline prior to OECs transplantation can reduce the cavity volume, astrogliosis, and the release of proinflammatory cytokines, providing unfavorable microenvironment and increasing the ability of OECs to enhance the axonal regeneration. According to the complexity of SCI pathophysiology, these results indicate that the combination therapy is more effective to improve SCI damage, and this study may be another promising step to the development of a combined treatment for refining the functional recovery after spinal cord injury. 
